# Activation of Sonic Hedgehog Signaling Promotes Differentiation of Cortical Layer 4 Neurons via Regulation of Their Cell Positioning

**DOI:** 10.3390/jdb10040050

**Published:** 2022-11-25

**Authors:** Koji Oishi, Kazunori Nakajima, Jun Motoyama

**Affiliations:** 1 Graduate School of Pharmaceutical Sciences, The University of Tokyo, Tokyo 113-0033, Japan; 2 Organization of Advanced Research and Education, Doshisha University, Kyoto 610-0394, Japan; 3 Department of Anatomy, Keio University School of Medicine, Tokyo 160-8582, Japan; 4 Laboratory of Developmental Neurobiology, Graduate School of Brain Science, Doshisha University, Kyoto 610-0394, Japan

**Keywords:** cerebral cortex, cortical subtype, layer, sonic hedgehog

## Abstract

Neuronal subtypes in the mammalian cerebral cortex are determined by both intrinsic and extrinsic mechanisms during development. However, the extrinsic cues that are involved in this process remain largely unknown. Here, we investigated the role of sonic hedgehog (Shh) in glutamatergic cortical subtype specification. We found that E14.5-born, but not E15.5-born, neurons with elevated Shh expression frequently differentiated into layer 4 subtypes as judged by the cell positioning and molecular identity. We further found that this effect was achieved indirectly through the regulation of cell positioning rather than the direct activation of layer 4 differentiation programs. Together, we provided evidence that Shh, an extrinsic factor, plays an important role in the specification of cortical superficial layer subtypes.

## 1. Introduction

The mammalian neocortex consists of two major types of neurons according to their usage of neurotransmitters. The majority of them are glutamatergic excitatory neurons derived from the dorsal telencephalon [1,2]. The other population is GABAergic inhibitory interneurons produced by the ventral telencephalon, which consist of 20–30% of all cortical neurons [3,4]. Both excitatory and inhibitory neurons can be further divided into a wider variety of types, which are recognized as neuronal subtypes, according to criteria other than neurotransmitters, such as morphology and gene expression profiles [5,6].

In the dorsal telencephalon, almost all subtypes of glutamatergic neurons are produced by common progenitor cells or neural progenitor/stem cells (NPCs) residing in the dorsal ventricular zone (VZ), which also give rise to glial cells including astrocytes and oligodendrocytes during late embryonic and early postnatal periods [7,8]. In the neurogenic period, NPCs sequentially generate different subtypes of neurons, which ultimately align in the cortical plate from the bottom to top parallelly to the pial surface and form cortical layers comprising anatomically distinguishable 6 layers [9]. Recent studies using unbiased approaches suggested that there are dozens of recognizable subtypes of cortical glutamatergic neurons in the motor area [10].

A plethora of efforts in recent years has provided evidence that determination of cortical subtypes is intrinsically regulated by specific transcription factors, such as Fezf2 [11,12,13], Bcl11b (aka Ctip2) [14], Rorb [15,16], Tbr1 [17], Brn1/2 [16], and Satb2 [18,19]. As subtype-specific features are established under the control of these factors, these factors are often called master regulators or subtype determinants. Given that NPCs sequentially generate different subtypes, temporal changes of NPC potentials, such as expression changes of subtype determinants, which actually occur in Drosophila NPCs [20], have been postulated [21]. However, many subtype determinants start to be expressed in postmitotic neurons, although some are also expressed in NPCs [12,22,23], leaving the question of what mechanism regulates the sequential generation of different subtypes from NPCs.

Not only intrinsic factors but also extrinsic factors, such as extracellular environments, play important roles in determining cortical neuronal subtypes. We and others have provided evidence that the specification of L4 neurons is controlled by extracellular environments [24,25,26,27]. Moreover, fate regulation by extracellular environments could occur more generally than previously thought [25,28]. However, given that these results were obtained mostly from transplantation experiments, molecular mechanisms that underpin this notion remain to be determined.

As such environmental cues, sonic hedgehog (Shh) is a strong candidate. On top of its role in the patterning formation and resulting specification of ventral structures in the CNS, Shh signaling regulates a wide variety of biological processes [29], such as the proliferation of intermediate progenitor cells [30], induction and expansion of outer radial glial cells that compose the outer SVZ, a progenitor pool commonly observed in the gyrencephalic neocortex [31], and gliogenesis [32,33,34]. However, the role of Shh signaling in the specification of cortical subtypes has remained unknown; cortical subtype phenotypes observed in Shh signaling mutants are mostly attributable to alteration in dorsoventral patterning and progenitor proliferation [35]. Given that specification of cortical subtypes, especially the L4 subtype, utilizes environmental cues [24,25,26,27], we investigated the role of Shh signaling in L4 subtype generation.

## 2. Materials and Methods

### 2.1. Mice

Pregnant ICR mice were purchased from Japan SLC (Shimizu laboratory supplies, Kyoto, Japan). The morning of vaginal plug detection was designated as E0.5. Mice were maintained on a 12 h light/dark cycle with free access to food and water. All experiments were approved by the Doshisha University Animal Experiment Committee and conducted in accordance with guidelines established by the Doshisha University Ethics Review Committee.

### 2.2. In Utero Electroporation

Pregnant mice were deeply anaesthetized, and in utero electroporation was carried out as described previously [36]. In brief, an empty or Shh-encoding plasmid vector together with the pCAGGS vector carrying the enhanced GFP cDNA (1 mg/mL) was injected into the lateral ventricle of the intrauterine embryos, and electronic pulses (33 V, 50 ms, 4 times) were applied using an electroporator (CUY21 EDIT II, BEX, Tokyo, Japan) with a forceps-type electrode (CUY650P5, Nepagene, Chiba, Japan).

For expression of Shh, the gene-encoding, full-length mouse Shh obtained from mouse cDNA was cloned into the plasmid vector pCAGGS or pEF.

### 2.3. Immunohistochemistry

Brains removed from embryos and pups were fixed for 1 h in phosphate-buffered saline (PBS) containing 4% PFA (*w*/*v*), incubated overnight at 4 °C with 20% sucrose in PBS (*w*/*v*), embedded in OCT compound (Sakura Finetek, Torrance, CA, USA), and sectioned with a cryostat to obtain 14 µm-thick coronal sections.

For primary antibodies, we used chick antibody to EGFP (Abcam, Cambridge, UK, ab13970), mouse antibody to Rorb (Perseus Proteomics, Tokyo, Japan, N7927), goat antibody to Lhx2 (Santa Cruz, sc-19344), mouse antibody to Brn2 (Santa Cruz, Dallas, TX, USA, sc-393324), and rabbit antibody to Shh (Santa Cruz, c-9024). For some cases, antigen retrieval was performed by incubating the sections for 20 min at 80 °C in 0.01 M sodium citrate buffer (pH 6.0). Because EGFP fluorescence disappeared by the antigen retrieval treatment, EGFP was immunostained with chick antibody against EGFP for revisualization. Immune complexes were detected with Alexa Flour-conjugated secondary antibodies (Invitrogen, Waltham, MA, USA). For nuclear staining, 1 µg/mL Hoechst 33,342 (Invitrogen) was used. Images were acquired using a confocal microscope (SP8, Leica, Wetzlar, Germany).

### 2.4. Quantitative Analysis of the Cell Positioning

To quantify the pattern of migration, the position of each GFP-positive cell relative to the total distance from the bottom of L4 or the subplate to the outer edge of the cortical plate (pial surface) was measured using the Image J software (National Institutes of Health shareware program), followed by sorting into 5 or 10 bins.

### 2.5. Statistical Analysis

Unless indicated otherwise, data are represented as means ± SEM of values from at least three embryos. For quantification of in vivo cell counting, all EGFP-positive cells were counted in the regions where rostrocaudal and mediolateral levels were carefully matched between animals. A representative section per electroporated embryo was quantified. The number of embryos analyzed was indicated in the figure legends. For two-group comparisons with equal variance as determined by the *F*-test, an unpaired Student’s *t*-test was used. Welch’s correction was used for unpaired t-tests of normally distributed data with unequal variance. Differences between groups were considered to be significant at *p* < 0.05. Each *p*-value was stated in figures or figure legends.

## 3. Results

**Figure 1:**
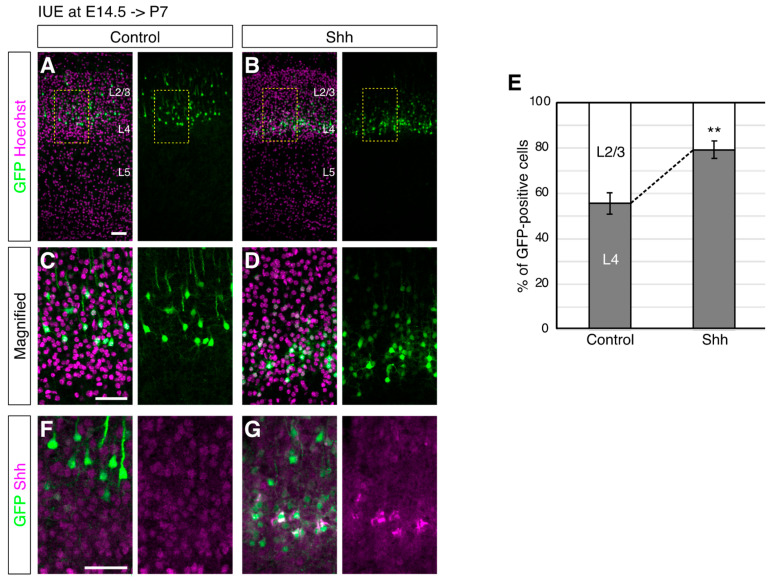
Shh overexpression at E14.5 increases the percentage of neurons in L4 at P7. (**A**,**B**). Empty (Control, (**A**)) or Shh expression vector (Shh, (**B**)) together with a GFP vector was electroporated into E14.5 brains, and then P7 brains were analyzed. The sections were counterstained with Hoechst (magenta). The boxed regions are shown at higher magnification in (**C**,**D**). (**E**). The percentages of the cells in L2/3 and L4 were determined in each condition. Quantitative data are presented (*n* = 3 for each group). ** *p* < 0.01. Note that the GFP-positive cells with Shh expression vector are located more in L4 than the control cells. (**F**,**G**). Expression of Shh was shown in the neurons treated as in (**A**,**B**). (Scale bars: 200 µm in (**A**,**C**,**F**)).

**Figure 2:**
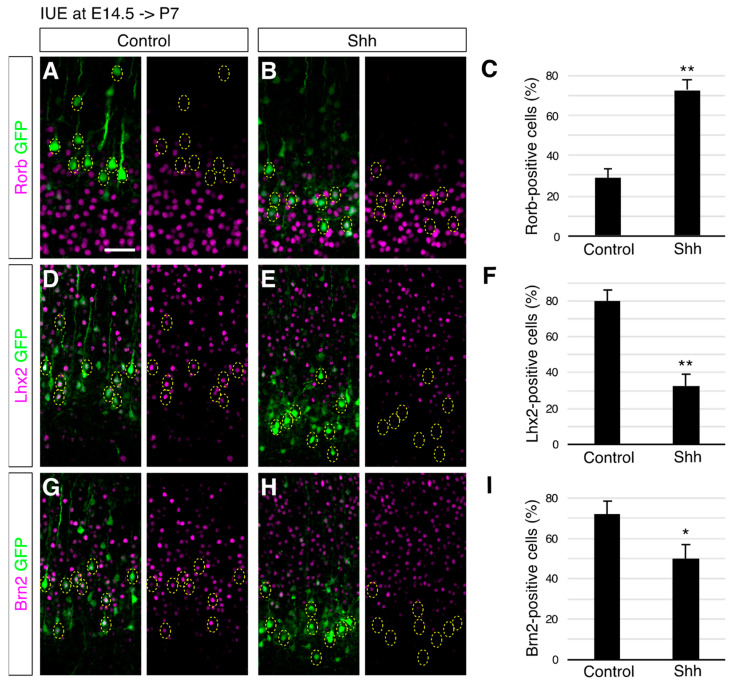
Shh-overexpressing cells at E14.5 acquire L4 characteristics. Empty (Control) or Shh expression vector (Shh) together with a GFP vector was electroporated into E14.5 brains, and then P7 brains were analyzed. The sections were immunostained for Rorb (**A**,**B**), Lhx2 (**D**,**E**), and Brn2 (**G**,**H**). The results of quantitative analysis for Rorb (**C**), Lhx2 (**F**), and Brn2 (**I**) are presented (*n* = 3 for each group). ** *p* < 0.01, * *p* < 0.05. Note that Shh-overexpressing cells acquired expression of Rorb, but lost expression of Lhx2 and Brn2. (Scale bar: 200 µm in (**A**)).

According to the previous implication that the specification of L4 neurons may require environmental cues [24,25,26,27], we first investigated the role of Shh signaling in L4 subtype generation in mice. We chose to manipulate Shh signaling by introducing a Shh expression vector in NPCs at embryonic day (E) 14.5 by in utero electroporation (IUE) [36] because NPCs at this stage give rise to both L4 and L2/3 neurons. In fact, 55.6% of the EGFP-labeled cells at E14.5 were located in the upper part of L4 at postnatal day (P) P7, while 44.4% of them were located in the lower part of L2/3 in the controls, where only an EGFP vector was introduced (Figure 1A,E). On the other hand, when an Shh expression vector together with an EGFP expression vector was introduced, the electroporated cells were located more in L4 (79.3% in the upper part of L4 and 20.7% in the lower part of L2/3, Figure 1B,E). The expression of ectopic Shh was detected around the EGFP-positive cells, suggesting autocrine action (or short distance effect) of ectopic Shh (Figure 1F,G). Accordingly, we did not observe obvious differences in the overall thickness of L2/3 and L4 (Figure 1 and Figure 2) although we cannot rule out the possibility of a non-cell-autonomous effect.

The observed phenotype could be attributable not only to migration or positioning failure but also to cell identity alteration. To distinguish these possibilities, we investigated the neuronal morphology, which often represents subtype-specific features [1,37], upon ectopic Shh expression. Magnified images showed that Shh overexpression decreased the neurons with a pyramidal shape that harbors an apical dendrite, a feature of L2/3 neurons, compared to those with a nonpyramidal shape, a feature of L4 neurons (Figure 1C,D) [15,16], suggesting that elevated Shh signaling modulates not only the positioning of superficial layer neurons but also their fate. These results suggest that elevated Shh signals enhance the generation of L4 neurons.

To further investigate the identity of the neurons with elevated Shh expression, we analyzed the expression of molecular markers that distinguish L4 and L2/3 neurons. An immunohistochemical analysis for Rorb, an L4 subtype marker [38,39], revealed that 28.6% of the control cells electroporated at E14.5 differentiated into Rorb-positive neurons at P7 (Figure 2A,C). In contrast, 72.3% of the Shh-overexpressing cells became Rorb-positive (Figure 2B,C). Moreover, the percentage of neurons that expressed L2/3 markers, such as Lhx2 [40] and Brn2 [23,41,42], was decreased by ectopic Shh expression (Lhx2, 80.2% in control, 32.7% in Shh overexpressed (Figure 2E,F); Brn2, 72.3% in control, 50.2% in Shh overexpressed (Figure 2G–I)). These results suggest that elevated Shh signaling promotes L4 generation, at the expense of L2/3 neurons, at molecular levels. This observation implied a rather surprising scenario, in which high levels of Shh signaling reversed the sequence of subtype specification of cortical neurons (L6- > L5- > L4- > L2/3) [8,9]; elevated Shh can change the ultimate identity of neurons that are destined to become L2/3 neurons into an L4 fate, an earlier-born subtype than L2/3 subtypes.

**Figure 3:**
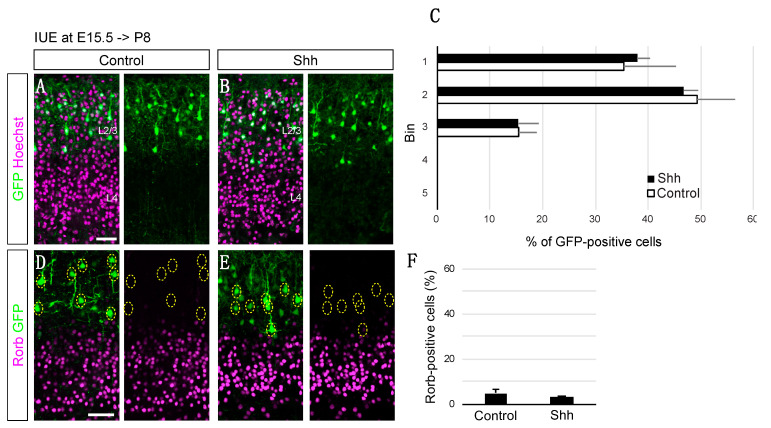
Shh overexpression at E15.5 does not alter cell positioning and Rorb expression. Empty (Control) or Shh expression vector (Shh) together with a GFP vector was electroporated into E15.5 brains, and then P8 brains were analyzed. The sections were counterstained with Hoechst (**A**,**B**) or immunostained for Rorb (**D**,**E**). (**C**). Quantitative data of cell positioning are presented. The position of each GFP-positive cell relative to the total distance from the bottom of L4 to the outer edge of the cortical plate was measured, followed by sorting into 5 bins (Control, *n* = 3; Shh, *n* = 4). (**F**). The result of quantitative analysis for Rorb is presented (Control, *n* = 3; Shh, *n* = 4). (Scale bars: 200 µm in (**A**,**D**)).

To test the possibility of juvenilization of NPCs by high levels of Shh signaling, we overexpressed Shh in later NPCs (IUE at E15.5), which produced predominantly L2/3 neurons and analyzed if they produced earlier-born neurons, such as L4 neurons. Cell positioning analysis showed that Shh overexpression at E15.5 did not change the ultimate positioning of the electroporated cells (Figure 3A–C). In addition, an immunohistochemical analysis for Rorb revealed that only a small fraction of E15.5-electroporated neurons expressed Rorb with or without the ectopic expression of Shh (Figure 3D–F). These results indicate essentially no generation of L4 neurons from E15.5-electroporated cells even in high levels of Shh signaling, suggesting that Shh does not generally regulate the temporal production of different cortical subtypes. In contrast, Shh would play a role in the demarcation of L2/3 and L4 neurons in a rather specific temporal manner.

As one of the mechanisms that determines L2/3 and L4 identity, we previously reported a cell position-dependent model, where L2/3 and L4 differentiation occurs along the ultimate positioning of the neurons in the superficial layer despite their birthdates [24]. Therefore, we hypothesized that Shh controls the cell positioning of E14.5-generated neurons. In this scenario, neurons that receive high Shh signals position the lower part of the superficial layer, where further differentiation processes occur.

**Figure 4:**
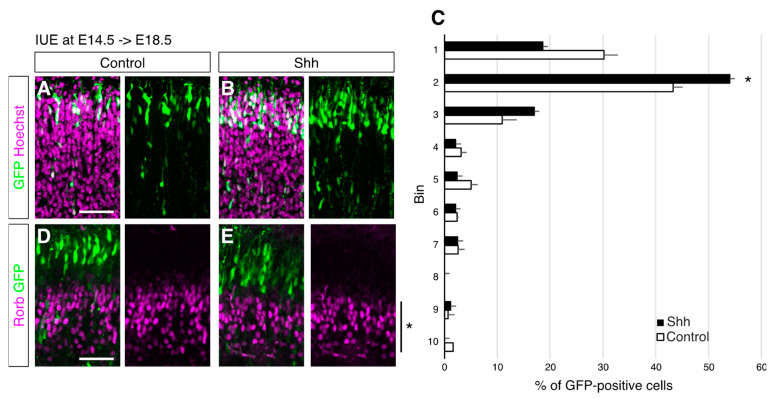
Upregulated Shh signaling regulates cell positioning without affecting an L4 marker expression. Empty (Control) or Shh expression vector (Shh) together with a GFP vector was electroporated into E14.5 brains, and then E18.5 brains were analyzed. The sections were counterstained with Hoechst (**A**,**B**) or immunostained for Rorb (**D**,**E**). The asterisk shows expression of Rorb in developing L5 neurons [16]. (**C**). Quantitative data of cell positioning are presented. The position of each GFP-positive cell relative to the total distance from the bottom of the subplate to the outer edge of the cortical plate was measured, followed by sorting into 10 bins (*n* = 4 for each group). * *p* < 0.05. (Scale bars: 200 µm in (**A**,**D**)).

We then asked if Shh regulates the positioning of neurons before affecting the cell differentiation status. We examined the brains at E18.5, when most of the E14.5-electroporated cells had reached the pial side of the cortical plate and started maturating. We found that Shh-overexpressing cells were located in deeper regions than the control cells at this early time point (Figure 4A–C). Since normal ‘future’ L4 neurons (that are destined to become L4 neurons) reach the pia surface earlier than future L2/3 neurons and later align deeper regions, this observation suggested that cell positioning/migration was already affected at this stage. On the other hand, the expression of Rorb was not detected in the electroporated neurons even with or without the ectopic expression of Shh (Figure 4D,E). These results suggest that upregulated Shh signaling can control the positioning of neurons before affecting their subtype identity.

**Figure 5:**
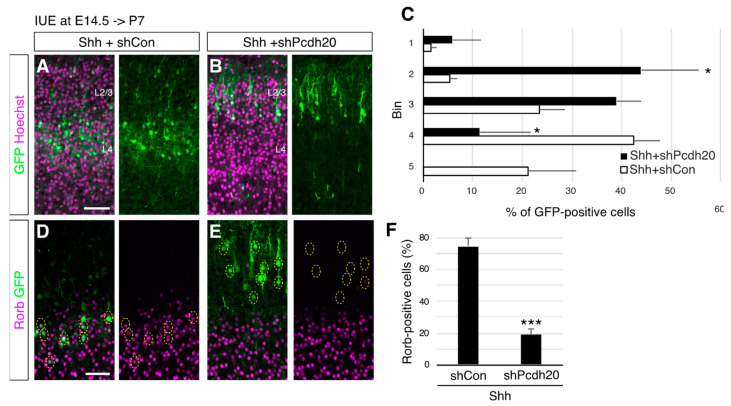
Shh overexpression promotes L4 fate acquisition in a cell positioning-dependent manner. Shh expression vector together with a control shRNA (Shh+shCon) or Pcdh20 shRNA vector (Shh+shPcdh20) was electroporated into E14.5 brains, and then P7 brains were analyzed. The sections were counterstained with Hoechst (**A**,**B**) or immunostained for Rorb (**D**,**E**). (**C**). Quantitative data of cell positioning are presented. The position of each GFP-positive cell relative to the total distance from the bottom of L4 to the outer edge of the cortical plate was measured, followed by sorting into 5 bins in each condition (*n* = 4 for each group). (**F**). The result of quantitative analysis for Rorb is presented (*n* = 4 for each group). * *p* < 0.05, *** *p* < 0.001. (Scale bars: 200 µm in (**A**,**D**)).

The proposed mechanism, in which Shh promotes L4 subtype generation via neuronal positioning, predicts that the neurons that fail to position the lower part of the superficial layer do not differentiate L4 neurons even if they receive high Shh signaling. To directly test this possibility, we tried to reposition the Shh-overexpressing cells back to the upper part of the superficial layer by the knockdown of Pcdh20, which changed the positioning of future L4 neurons into more upper regions without affecting neuronal migration and early subtype specification [24]. We found that Pcdh20 knockdown was able to reposition Shh-overexpressing neurons back to more superficial regions (Figure 5A–C). We then investigated the expression of Rorb in these cells. Rorb staining revealed that 74.6% of Shh-overexpressing neurons were positive for Rorb, but this percentage was reduced to 18.9% by simultaneous Pcdh20 knockdown (Figure 5D–F). These results strongly support the notion that high levels of Shh signaling promote the specification of an L4 subtype via cell positioning.

## 4. Discussion

In this study, we found a potential role of Shh signaling in the generation of L4 subtypes of the mouse cortical plate. Shh signaling appeared not to directly activate L4 specification programs but controlled the positioning of a subset of superficial layer neurons, thereby leading to the ultimate specification of L4 subtypes. A similar regulation of cell positioning by Shh signaling was reported previously in the chick optic tectum [43], suggesting a wider role of Shh in cell positioning/distribution.

Shh has been shown to play roles in the proliferation and cell cycle control of progenitor cells in both positive and negative ways during neurogenesis [29]. We previously reported that ectopic expression of Shh in developing NPCs resulted in an increased proliferation of intermediate progenitor cells [30]. As the method used in the current study is similar to that in the previous one, high levels of Shh signaling might have also increased the proliferation rate of intermediate progenitor cells in the present study. However, although increased proliferation may increase the generation of later-born subtypes, altered proliferation did not account for the observed phenotypes that high levels of Shh signaling led to the generation of ‘earlier-born’ subtypes than the control.

What downstream effectors play a role in this type of subtype specification? Upon binding to its receptors, Shh influences a wide variety of signal transduction pathways including the activation of the transcription factor Gli1 [44]. It is well studied that Shh determines an oligodendrocyte fate through Gli1-dependent transcriptional regulation [45,46]. Gli1 directly upregulates Olig2, a master regulator of oligodendrocytes, allowing NPCs to differentiate into oligodendrocytes [45]. Thus, a similar mechanism, by which Shh determines the L4 subtype via direct transcriptional regulation, such as upregulation of L4 fate determinants, is conceivable. However, we are not in favor of this hypothesis due to mainly three reasons. First, we did not observe a premature expression of Rorb in early time points; if Shh directly activated the L4 specification program, the premature induction of downstream targets would be predicted. Second, the repositioning of Shh-overexpressing neurons to the more pial side in the superficial layer canceled the expression of Rorb even in the presence of Shh. Third, Gli1 transcriptional activation, a canonical downstream target of the Shh–Ptch1 axis [29,44], was hardly detected in the developing cortical neurons [47]. Accordingly, we did not detect *Gli1* and *Ptch1* mRNAs even in Shh-overexpressing neurons. Instead, another Shh receptor, Boc, which activates noncanonical Shh pathways, was strongly expressed [48]. These observations suggest that Shh may indirectly regulate the generation of L4 subtypes.

It remains to be determined what kind of intracellular events Shh signaling regulates to control cell positioning. A possible downstream is calcium signaling [49], which has been shown to play a role in the regulation of neuronal migration [50,51]. In addition, we recently found that Shh can activate calcium signaling (J.M. unpublished observation) [52], leading to a hypothesis that the Shh–calcium axis controls neuronal migration and positioning.

It is also to be determined how Shh specifically regulates L4 development. Shh may act as a limiting factor for immature neurons to be located in the future L4 region (bottom of the superficial layer); the amount of Shh is not abundant so that only a part of E14.5-born cells can receive Shh signaling, which accelerates the positioning of neurons in the future L4 region, where further L4 maturation processes occur. The neurons that do not receive enough Shh are positioned in the more superficial or future L2/3 region and differentiate into L2/3 subtypes. Such endogenous Shh might be provided from the marginal zone of the outermost cortical region, where cortical interneurons are migrating. In fact, interneurons were reported as one of the Shh sources in the developing cortex [53]. Ectopic Shh may have activated the population that normally does not receive Shh signaling, enabling them to be located in the future L4 region. Such differentiation plasticity is probably also regulated temporally in NPCs and/or immature neurons because ectopic Shh expression did not cause any alteration of cell positioning and subtype specification in the E15.5-electroporated cells. As Boc is expressed strongly in L4 (formed by mainly E14.0-born neurons) but very weak in L2/3 (formed by E15.5-born neurons), Boc expression levels could underlie this differential response to ectopic Shh [48]. In addition, given that E14.5-born neurons can respond to ectopic Shh, one might expect that Boc-high neurons exist in layer 2/3, presumably at its bottom. Further studies on the detailed expression pattern of Boc will clarify the difference.

Although Reelin, an extracellular protein, is well-known as a factor that controls positioning and/or migration of cortical neurons [54], cortical lamination has been regarded as a relatively intrinsic process, in which new neurons just pile up on the earlier-formed ‘layers’ according to their birthdates. Therefore, the extent to which the lamination process is regulated by extrinsic factors remains obscure [5]. Here, we reported on Shh as an extracellular regulator in the lamination of excitatory cortical neurons. A recent report showing the involvement of Cxcl12 in the positioning of cortical interneurons [55] would predict further roles of extrinsic cues in the regulation of neuronal migration/positioning and lamination.

## Data Availability

Any data and original materials are available from the corresponding author upon reasonable request.
